# Phenotypic diversity and selection maintain *Leishmania amazonensis* infectivity in BALB/c mouse model

**DOI:** 10.1590/0074-02760160280

**Published:** 2017-01

**Authors:** Benoît Espiau, Virginia Vilhena, Armelle Cuvillier, Aldina Barral, Gilles Merlin

**Affiliations:** 1 Université de Bordeaux, Laboratoire de Génomique Fonctionnelle des Trypanosomatidés, Bordeaux, France; 2 LabEx Corail, Papetoai, Moorea, Polynésie Française; 3 Fundação Oswaldo Cruz, Instituto Gonçalo Moniz, Salvador, BA, Brasil; 4 Faculdade Anhanguera de Brasília, Brasília, DF, Brasil; 5 B Cell Design, Limoges, France; 6 Instituto de Investigação em Imunologia, Salvador, BA, Brasil

**Keywords:** Leishmania amazonensis, subcloning, phenotypic diversity, infectivity, selection

## Abstract

*Leishmania* are protozoan parasites that show remarkable diversity, as revealed by the various clinical forms of leishmaniasis, which can range from mild skin lesions to severe metastatic cutaneous/mucosal lesions. The exact nature and extent of *Leishmania* phenotypic diversity in establishing infection is not fully understood. In order to try to understand some aspects of this diversity, we subcutaneously infected BALB/c mice with first and second generation subclones of a *L. amazonensis* strain isolated from a patient (BA125) and examined in vivo lesion growth rate and antimony susceptibility. In vivo fast-, medium- and slow-growing subclones were obtained; moreover, fast-growing subclones could generate slow-growing subclones and inversely, revealing the continuous generation of diversity after passage into mice. No antimony-resistant subclone appeared, probably a rare occurrence. By tagging subclone cells with a *L. amazonensis* genomic cosmid library, we found that only a very small number of founding cells could produce lesions. *Leishmania* clones transfected with in vivo selected individual cosmids were also diverse in terms of lesion growth rate, revealing the cosmid-independent intrinsic characteristics of each clone. Our results suggest that only a few of the infecting parasites are able to grow and produce lesions; later, within the cell mixture of each lesion, there coexist several parasite populations with different potentialities to grow lesions during the next infection round. This may reflect a sort of programmed heterogeneity of individual parasites, favoring the survival of some individuals in various environmental conditions.

*Leishmania* are digenetic protozoan parasites of the Trypanosomatidae family that are responsible for severe diseases in tropical and subtropical countries. Discovered more than a century ago, the parasites exist in two forms: the amastigotes that live inside parasitophorous vacuoles of mammalian macrophages, and the promastigotes that live extracellularly in the lumen of the digestive tract of phlebotomine sandflies.

There are different forms of leishmaniasis, and *Leishmania* cells appear to develop in vitro resistance to most drugs relatively easily under selection. Several studies have shown that isolates and strains of these parasites are heterogeneous and polymorphic in the wild and in the laboratory, in terms of pathogenicity and genomic organisation. It is still difficult to associate virulence/host immune response/clinical form of the disease to particular genomic characteristics (Greenblatt et al. 1990, Kahl et al. 1990, Almeida et al. 1996, Noyes et al. 1997, Cupolillo et al. 1998, El Tai et al. 2001, Schonian et al. 2001, Victoir & Dujardin 2002). To further complicate the situation, the same parasite strain does not cause the same disease in different strains of mice (Kahl et al. 1990, Martinez et al. 1991, Sinagra et al. 1997, Sulahian et al. 1997, Sakthianandeswaren et al. 2009). Differences have even been described between isolates of the same patient (Cuervo et al. 2004) or clones of the same *Leishmania* species, as cloning of a virulent strain could generate virulent and avirulent clones (Handman et al. 1983, Garin et al. 2001). Diversity in virulence among clones of a metastatic *L. guyanensis* strain (Martinez et al. 2000) and in antimony susceptibility among isolates from patients and clones recovered from these isolates (Moreira et al. 1998, Carrio et al. 2000, Pal et al. 2001) have been described. Moreover, isolates from antimony unresponsive patients have been shown to contain a mixture of susceptible and resistant clones (Bhattacharyya et al. 2002).

With the aim to study *Leishmania* phenotypic diversity, we chose an in vivo infection animal model, simplified compared to natural infections, but with the advantages of some parameter constancy. Our model consisted in an infectious strain of *L. amazonensis* isolated from a patient (BA125), the BALB/c mouse strain, and the footpad infection. Two criteria of clinically relevant potential diversity were examined, *i.e.*, in vivo lesion growth rate and antimony susceptibility. We minimised and standardised as much as possible the unavoidable in vitro culture steps, as they are known to introduce uncontrollable effects on the infectivity stability. First, cells extracted from a parental lesion were subcloned and individual subclones used for new infections in mice treated or not with antimony; the cells of some of the obtained lesions were again subcloned as for the first sets. Then the parental strain was transfected with a *L. amazonensi*s cosmid genomic library, mice infected with the transfectants, and the cosmids recovered from the lesion cells. Lesion growth kinetics, phenotype stability and cosmid occurrence in recovered lesion amastigotes were compared.

*Leishmania* infectivity depends upon multigenic determinants (Chang et al. 2003) and the precise cause of its loss or persistence is not really understood. The purpose of this report is not to address this complex question, but to describe in more detail a biological phenomenon of interest and propose an interpretation. We hypothesize that (i) among the cells used for infection, only a few are able to grow and produce lesions and (ii) different cells with different potentialities to grow lesions during the next infection round coexist in the cell mixture of each lesion.

## MATERIALS AND METHODS

*Parasite culture* - The strains used in this study were *L. amazonensis* MHOM/BR/1987/BA125 (BA125) and MHOM/BR/1987/BA276 (BA276). Promastigotes were cultured as described previously (Cuvillier et al. 2000) at 24ºC in AM medium, *i.e.* RPMI-1640 medium plus 25 mM Hepes pH 7.5, 2 mM glutamine, 2 mg/mL dextrose, 1 mM sodium pyruvate, 1x MEM essential and non-essential amino acids, 50 units/mL penicillin, 50 mg/mL streptomycin, and 8% FCS (all ingredients were purchased from Eurobio) in closed flasks under gentle agitation. The cells were passaged every two-three days in order to keep the cell density between 4 x 10^6^ and 1.5 x 10^7^ cells per mL.

*L. amazonensis subcloning* - Promastigotes were subcloned by using two different methods. Method I: exponentially growing cells were diluted to a density of 10^3^ cells per mL; 0.5-mL drops of the suspension were loaded into 96-well microtiter plates and carefully inspected visually under an inverted microscope (ocular 10X, lens 40X); only wells containing a single live promastigote were supplemented with additional 100 mL AM medium; microtiter plates were then incubated at 24ºC with CO2; subclones (about one out of 10 drops) were cultured until they reached the number required for making stabilates and infecting mice (about 6-7 weeks). Method II: 0.5 mL of exponentially growing cells, diluted to 20-50 cells per mL, were spread onto 10-cm Petri dishes containing 0.7% SeaPlaque agarose (low-melting, BMA) in AM medium; dishes were pre-dried for 1 h under a sterile hood, caps off, and subsequently incubated for 2-3 weeks at 24ºC with CO2; when colonies became visible by eye, they were transferred to 96-well microtiter plates containing 100 mL AM medium and further cultivated as in Method I.

*Experimental infection of mice and antimony treatment* - *L. amazonensis* stationary phase promastigotes (five days after last passage, density of 1.5 to 1.8 x 10^7^ cells per mL) were harvested, washed twice with phosphate-buffered saline (PBS), counted and resuspended in PBS at 5 x 10^8^ cells per mL; 5 x 10^6^ cells (*i.e.* 10 mL) were injected subcutaneously into the hind footpads of five-week-old female Balb/cJRj mice (Centre d’Elevage Robert Janvier). Antimony-treated mice were i.p. injected every day following infection, for two weeks, with 0.1 mL Glucantime™ (Specia). Footpad size was monitored once every week using a caliper. Each mouse was tagged with distinctive ink marks for individual recognition.

*Recovery of Leishmania cells from lesions* - When lesions reached 8 to 10 mm diameter, mice were sacrificed; the infected footpads were cut off, plunged into ethanol for 10 min; then, lesions were macerated in PBS, cell aggregates homogenised in a Thomas potter, and debris eliminated by low-speed centrifugation; amastigotes remaining in the supernatant were washed twice with PBS and further dissociated by repeated passages through 1-mL syringes with 23G/26G needles. Amastigotes were counted, transferred to AM medium at 2-5 x 10^6^ cells per mL and incubated at 24ºC. 48 h later, newly differentiated promastigotes were cloned as described above.

*Subclone tagging with a cosmid library of L. amazonensis* - We used the previously described genomic *L. amazonensis* BA276 DNA cosmid library (Lachaud et al. 2014) to tag the *Leishmania* clones; this library was constructed with the pcosTL vector, which confers G-418 resistance (Kelly et al. 1994), and propagated in the *Escherichia coli* STBL-2™ strain (Invitrogen); it contains about 20,000 independent 50-kb insert clones, representing more than 15 haploid genome copies. 2,500 individual clones were kept at -80ºC in 96-well microtiter plates in LB medium plus 50% glycerol. Cosmid DNA was extracted from bacteria by using the Triton X-100 method (Ausubel et al. 1989). For cosmid DNA extraction from *Leishmania* amastigotes or promastigotes, 10^8-9^ cells were washed twice with PBS, resuspended in 0.5 mL 10 mM Tris-HCl pH 7.5, 0.25 mM NaCl, lysed by addition of 4.5 mL 10 mM Tris-HCl pH 8, 10 mM EDTA, 10 mM NaCl, 0.5% SDS, and incubated for 2 h at 50ºC with 0.5 mg Proteinase K; after two phenol/chloroform extractions, the DNA was ethanol precipitated, recovered with a Pasteur pipette, dissolved in 4 mL TE buffer (10 mM Tris-HCl, 1 mM EDTA), incubated with 0.1 mg RNAse A for 2 h at 37ºC, re-extracted twice with phenol/chloroform, ethanol precipitated, washed twice and recovered in 0.5 mL sterile TE buffer.

For *Leishmania* transformation, cosmid-containing bacterial clones were individually grown in 300 µL LB medium; about 400 cultures were then pooled and the cosmid DNA extracted. Each DNA pool was electroporated into *L. amazonensis* BA125 promastigotes (50 µg DNA, 10^8^ cells in 250 µL, 450 V, 74 Ω, 600 µF, 2-mm cuvettes) derived from footpad lesion amastigotes that were extracted 48 h before the electroporation. Transfected *Leishmania* cells were immediately spread onto 0.7% agarose plates containing AM medium plus 10 µg/mL G-418 (Cuvillier et al. 2000). Isolated *Leishmania* colonies were then picked and further grown individually. 400 transformed, five-day stationary phase *Leishmania* clones were then pooled and injected into the hind footpads of 6-12 BALB/c mice (10^6^ cells/footpad). Half of the mice were treated with Glucantime™ as described above and lesion growth monitored every week. Amastigotes were recovered from individual lesions. Cosmid DNA was extracted from amastigotes and 100 ng electroporated into 50 µL electrocompetent *E. coli* STBL-2™ bacteria (1600 V, 192 Ω, 40 µF, 1-mm cuvettes). Transformed bacterial colonies were picked and the cosmids they contained identified by *Sal I* restriction band pattern on 0.7% agarose gels.

## RESULTS

*Cloning the L. amazonensis BA125 strain* - This strain was isolated from a patient in 1987 and has since been grown in vitro and passaged into BALB/c mice for an undetermined number of times. Two months after an experimental infection, amastigotes were recovered from a 1-cm footpad lesion; 48 h later, newly differentiated promastigotes were subcloned using Method I or II (see Materials and methods). Groups of six BALB/c mice were infected in both hind footpads with stationary phase cells of one of fifteen randomly selected subclones or the parental line BA125. Three of the six mice were treated with antimony. The evolution of the lesion size was monitored during the following weeks.

**Fig. 1:**
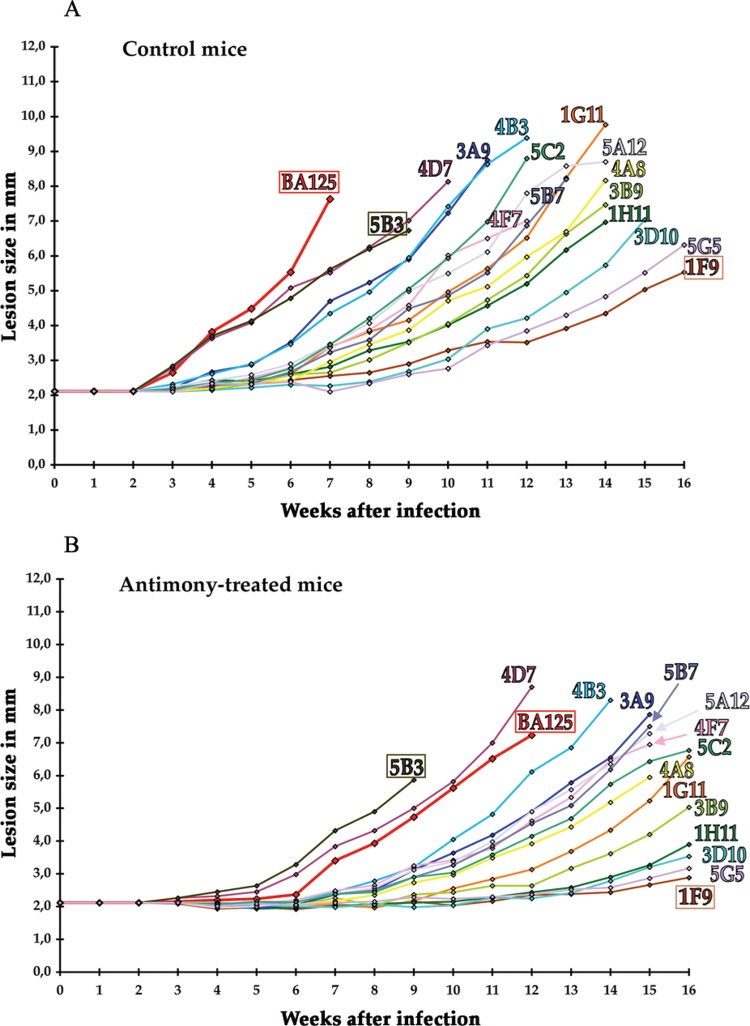
: growth kinetics of lesions generated by the parental BA125 strain of *Leishmania amazonensis* and its subclones. (A) Control mice; (B) antimony-treated mice (daily injections for the first two weeks after infection). Each group contained three mice, and both hind footpads of each mouse were infected; data are the mean value of the six footpad sizes. For clarity, the standard deviation (15-20%) is not indicated. Uninfected footpads kept a value of 2 mm during the time of the experiment (not shown). Mice were sacrificed when the lesions reached 8-10 mm.

Fig. 1 shows the results obtained with a set of subclones isolated using Method I. Growth kinetics in untreated mice revealed the existence of fast-growing (*e.g.*, 5B3), slow-growing (*e.g.*, 1F9) and intermediate (*e.g.*, 1G11) subclones (Fig. 1A). Although there was some diversity in lesion sizes, the standard deviation was usually less than 15% within a group. Thus, out of fifteen subclones, four grew very fast (5B3, 4D7, 3A9, 4B3), three rather slowly (3D10, 5G5, 1F9), and the others displayed an intermediate rate. In antimony-treated mice (Fig. 1B), lesion growth was uniformly slowed: the fastest subclones (5B3, 4D7) were still the fastest to grow; the slow-growing ones grew even slower (1F9, 5G5). The only effect of antimony treatment was to slow lesion growth.

**Fig. 2:**
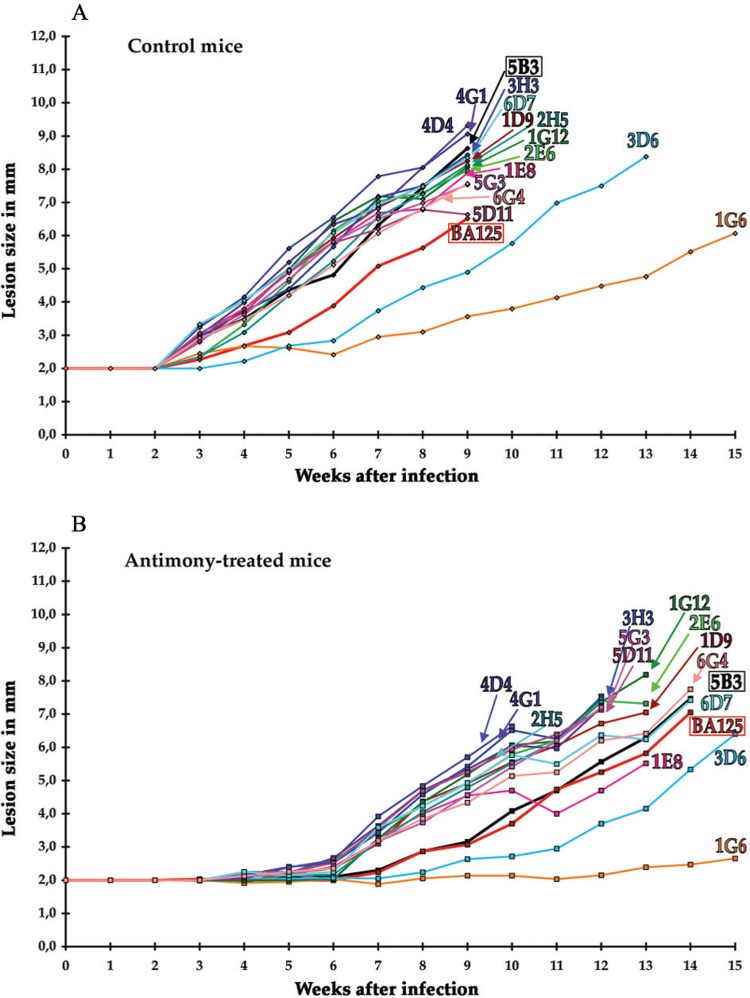
: lesion growth kinetics for subclones of a first generation fast-growing subclone (5B3 of Fig. 1). (A) Control mice; (B) antimony-treated mice. Red: parental BA125 strain.

Similar results were obtained with another set of fifteen subclones using cloning Method II. In both cases, however, no antimony-resistant subclone was found.

Thus, the cell population found in a lesion appeared heterogeneous in its capacity to grow lesion.

*Subcloning a fast- and slow-growing first generation subclone* - The stability of the phenotypes was investigated. Amastigotes from a single lesion developed by a fast-growing first generation subclone (5B3 of Fig. 1) were extracted, left to differentiate to promastigotes for 48 h, and subcloned by Method I or II. Then, groups of BALB/c mice were infected as above with these second generation subclones, and lesion growth monitored (Fig. 2). Twelve out of fourteen second generation subclones were fast-growing, like the parental first generation subclone 5B3, while two (3D6 and 1G6) were slow-growing (Fig. 2A, untreated mice). As previously (Fig. 1B), the antimony treatment only delayed growth for a few weeks (Fig. 2B). This experiment was repeated with another set of second generation subclones issued from another fast-growing first generation subclone; the same results were obtained (not shown). Thus, like the parental BA125 strain, a lesion generated by a fast-growing first generation subclone contained heterogeneous cells, at least regarding the growth rate of the lesion, as slow-growing cells were produced.

**Fig. 3:**
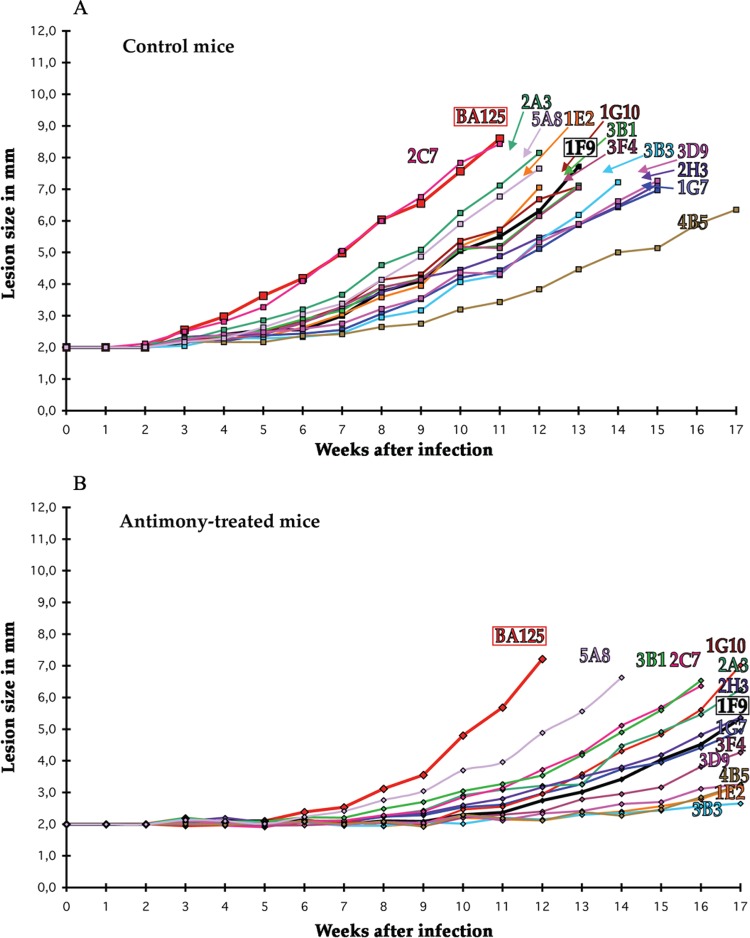
: lesion growth kinetics for subclones of a first generation slow-growing subclone (1F9 of Fig. 1). (A) Control mice; (B) antimony-treated mice. Red: parental BA125 strain.

The next question was: what happens with slow-growing subclones? Cells from a lesion generated by a slow-growing first generation subclone (1F9 of Fig. 1) were extracted, subcloned and used for footpad infection as above (Fig. 3). Most second generation subclones grew relatively slowly, like the parental subclone 1F9, and slower than the parental BA125 strain, except subclone 2C7 which grew as fast as the parental BA125 strain (Fig. 3A, untreated mice). This “reversion” occurred at a relatively high frequency (1/12). Concerning the antimony treatment (Fig. 3B) no resistant subclone was found; again, lesion growth rates were slowed, particularly for second generation subclones 2C7 and 1E2 which appeared hypersusceptible to antimony.

Thus, we observed the generation of diverse phenotypes with regard to lesion growth rate each time *L. amazonensis* BA125 parasites were passaged into mice. Antimony resistance was not found within our subclone library and may require a more continuous in vivo selection. The cell populations appeared as mixtures of phenotypically different cells and the phenotypes did not seem stable within a subclone. To confirm this, we attempted to trace the subclones within lesions.

*Restricted subclone diversity of L. amazonensis cells within lesions* - Tagging can be performed by using either a vector or a cosmid strategy. We first considered tagging the subclones by transfecting the parasites with a library of random non-coding DNA sequences inserted into the pTEX vector, which confers G-418 resistance (Kelly et al. 1992). However, the DNA extracted from *L. amazonensis* BA125 cells transfected with an empty pTEX plasmid was unable to transform bacteria (probably because the small episomes formed very large concatenates that could not enter the bacteria) (Kelly et al. 1992); with such strategy, it would have been impossible to analyse the episomes found in lesion amastigotes.

On the other hand, the DNA recovered from pcosTL (Kelly et al. 1994)-based cosmid-transfected *Leishmania* cells (that were G418-resistant) led to successful transformation of bacteria and DNA analysis of the recovered cosmids by *Sal I* restriction band pattern. Since a genomic DNA library of the *L. amazonensis* BA276 strain in cosmid pcosTL was available (Lachaud et al. 2014), we decided to use the cosmid strategy for the *Leishmania* clone tagging attempts.

In a first experiment, 384 individually grown bacterial cosmid clone cultures were pooled, the cosmids extracted and used to transform freshly differentiated *L. amazonensis* BA125 promastigotes. After selection of the transformants on agarose plates with G-418, 414 colonies were picked, individually grown, pooled and one million cells injected into hind footpads of six BALB/c mice. Two months later, when lesions had developed, cosmids were extracted from lesion amastigotes, reintroduced into *E. coli*, and cosmid DNA from 24 bacterial colonies analysed by *Sal I* restriction digestion for each of the six mice. To control the cosmid diversity of the transformed *Leishmania* pool, cosmids were also extracted from an aliquot, just before mice infection, and analysed. Before infection, 36 cosmid types were found out of 71 bacterial colonies, of which 21 were represented once, nine twice, three thrice, meaning that the expected diversity of cosmids was somehow respected (Table I). After infection, only three-nine cosmid types per mouse could be found out of 24 bacterial clones; besides, a few cosmid types were over-represented and common to several mice: cosmid T26 (36% of bacterial clones), cosmid T30 (15%), and cosmid T33 (15%). Remarkably, these cosmids were under-represented in the 71 bacterial colonies analysed before passage in mice: T26 was found only twice (3%), and cosmids T30 and T33 were not found at all. Thus, passage in mice led to an enrichment of a small number of cosmids.

**TABLE I:** The different types of cosmids found in a *Leishmania* pool before and after passage in mice

Cosmid types	Extracted from promastigotes^1^	Extracted from lesion amastigotes from antimony-treated mice						

		Number of occurrences in 24 bacterial clones						Total out of 144 clones

		Mouse 1	Mouse 2	Mouse 3	Mouse 4	Mouse 5	Mouse 6	
2 types (unnamed)	5	0	0	0	0	0	0	0
T42	4	0	0	0	1	0	2	3
T35	3	0	0	1	3	0	1	5
2 types (unnamed)	3	0	0	0	0	0	0	0
T26	2	16	11	5	13	1	6	52
T36	2	0	0	1	0	0	0	1
T37	2	0	0	0	1	0	0	1
6 types (unnamed)	2	0	0	0	0	0	0	0
21 types (unnamed)	1	0	0	0	0	0	0	0
T27	0	1	0	0	0	0	0	1
T28	0	1	0	0	0	0	0	1
T29	0	1	0	0	0	0	0	1
T30	0	3	11	3	0	0	5	22
T31	0	1	0	0	0	0	0	1
T32	0	1	0	0	0	0	0	1
T33	0	0	1	13	0	8	0	22
T34	0	0	1	0	0	0	0	1
T38	0	0	0	0	2	0	0	2
T39	0	0	0	0	2	0	0	2
T40	0	0	0	0	0	0	4	4
T41	0	0	0	0	0	0	1	1
T43	0	0	0	0	0	0	1	1
T44	0	0	0	0	0	2	3	5
T45	0	0	0	0	0	0	1	1
Unidentified	0	0	0	1	2	13	0	16

Total types		7	4	5	6	3	9	20

^1^: extracted from *Leishmania* pool promastigotes just before mice infection. Number of occurrences in 71 bacterial clones.

*Involvement of intrinsic factors for in vivo selection* - In another experiment, lesion amastigotes from antimony-treated mice were recovered and their cosmid content analysed (Table II). As previously, after 48 h a limited number of cosmid types were found: T3 (40%), T5 (30%), T8 and T9 (8%), plus several less represented ones (T11 and T15, 2% each) (Table II, column A). Interestingly, we also observed a loss of diversity during in vitro growth of the derived promastigotes: two months after seeding, cosmid T15 became predominant (41%) while T5 and T3 diminished (22 and 25% resp.), and four months later, there was 100% cosmid T15.

**TABLE II:** Cosmid diversity in in vitro culture

Cosmid types	Among bacterial clones (%)		

	A	B	C
		
	48 h after extraction from lesion^*a*^	Two months in culture^*b*^	Four months in culture^*c*^
T1	2	0	0
T2	2	0	0
T3	40	25	0
T4	0	4	0
T5	30	22	0
T6	4	0	0
T7	2	0	0
T8	8	0	0
T9	8	4	0
T10	0	4	0
T11	2	0	0
T15	2	41	100

*a*: 48 bacterial clones analysed; *b*: 24 bacterial clones analysed; *c*: 24 bacterial clones analysed.

The 50-kb genomic fragments of the cosmids could code for approximatively 10 genes each, and these genes could influence the in vivo selection, as they were probably expressed in the transfectant cells. To test this, four cosmids (T3, T5, T8 and T15), chosen for their different abundance in lesions, were transfected individually into freshly prepared promastigotes. Three independent colonies transfected by each cosmid were isolated on agarose plates, grown, injected into mice footpads, and the lesion growth monitored with and without antimony treatment (data not shown). Considering cosmid T3 for example, colony T3-1 grew lesions very slowly (similar to subclone 1F9 of Fig. 1), colony T3-3 grew lesions very fast (similar to subclone 5B3 of Fig. 1) and colony T3-2 was intermediate (similar to subclone 1G11 of Fig. 1), and all three were susceptible to antimony. Similar results were obtained with the other cosmids (T5, T8 and T15). In conclusion, different subclones transfected with the same cosmid displayed different phenotypes. At least in these experiments, the phenotypes reflected intrinsic properties of the subclones and were not cosmid-dependent.

*Evolution of pools of clones* - 16 mice were infected with a mixture of equal amounts of three very different subclones, T5-1 (fast lesion growth), T3-1 (slow lesion growth) and T15-4 (no detectable lesion after six months); half were treated with antimony, the other half left untreated. Cosmids were isolated from mature lesions of individual mice as previously. As shown in Table III, for control mice, cosmid T5 (of subclone T5-1, the fast growing one) represented 80-90% of the recovered cosmids, cosmid T3 (of subclone T3-1, the slow growing one), 10-20% and T15 (of subclone T15-4, the non-growing one), 0%. There were only slight variations from mouse to mouse and antimony treatment only slowed lesion growth without changing the proportions of the recovered plasmids.

**TABLE III:** In vivo outcome of mixtures of defined clones

*Leishmania* clones used for infection	Mouse treatment	Mouse #	Cosmids recovered from lesions (number of bacterial clones)				

			T3	T5	T15	?	Total
Equal amounts of T3-1 (slow growth), T5-1 (fast growth) and T15-4 (no lesion after six months)	Untreated	1	3	20	0	1	24
		2	3	21	0	0	24
		3	8	15	0	1	24
		4	5	19	0	0	24
		9	0	23	0	1	24
		10	1	22	0	1	24
		11	2	22	0	0	24
		12	2	22	0	0	24

	Treated	5	0	24	0	0	24
		6	2	22	0	0	24
		7	3	21	0	0	24
		8	4	20	0	0	24
		13	0	24	0	0	24
		14	8	15	0	1	24
		15	3	21	0	0	24
		16	3	21	0	0	24

In all, with regard to lesion growth, the *L. amazonensis* strain used consisted in a mixture of different cells, some able to develop lesions rapidly, some less rapidly. Whether this feature is reversible or not is not known; we only observed the presence of a minority of slow-growing cells in lesions that developed rapidly, and a minority of fast-growing cells in lesions that developed slowly. This diversity seemed to be recreated after each passage in mice. Only the cells originating from a few founding ones were present in the lesions. A similar restriction of diversity could be observed in in vitro culture. Whether this can be generalised or not to other *Leishmania* strains or species is unknown. Exploring these phenomena might prove useful for the study and understanding of *Leishmania* biology.

## DISCUSSION

In this work, we studied the infectivity of freshly isolated *L. amazonensis* clones and subclones from mouse footpad lesions. Regarding lesion growth rate, the cloned parasites were heterogeneous, as some grew faster than others. Such heterogeneity in the cell population of a strain that has been passaged in mice for years was somehow surprising, as one could have expected the non-infectious cells to have been eliminated. In fact, heterogeneity was found within subclones after each passage in mice. Besides, only a very small number of founding infecting parasites appeared responsible for lesion development.

Changes in *Leishmania* infectivity have been described long ago. Repeated in vitro passages of *Leishmania* lead to loss of infectivity (Giannini 1974, Nolan & Herman 1985), although some clones were reported to remain infectious for years (Greenblatt et al. 1985, Segovia et al. 1992). It has also been noted that infectivity could be restored after passage in animals (Katakura & Kobayashi 1985). Concerning *L. amazonensis*, the only available data report the loss of mouse infectivity of uncloned promastigotes with increasing periods of in vitro cultivation; it was related to a decreased N-glycosylation of surface proteins like gp-63 (Kink & Chang 1988). In our case, we reduced to a minimum the time promastigotes spent outside animals, usually six to seven weeks from lesion amastigote extraction to footpad infection, comprising promastigote differentiation, subcloning and amplification of the subclones. We standardised the infection procedure by using the same amount (five million) of promastigotes of the same age (five-day stationary phase). We also cultivated the parental strain in parallel during the cloning process such that it spent the same period of time in in vitro cultures as the subclones; in spite of these precautions, there was a certain variability in the lesion growth rate of the parental strain from an experiment to another, possibly due to different growth rates of infectious and non-infectious cells within the mixture, as suggested by other authors (Kink & Chang 1988); however, these variations appeared small compared to the differences between fast- and slow-growing subclones.

In order to tag subclones and be able to recognise their cell members within mixtures, we used cells transfected with unique cosmid episomes. This method is, however, limited for at least two reasons.

Firstly, maintaining 100% episomes inside *Leishmania* cells requires a selection pressure, in our case G-418. Without antibiotic, episomes are progressively lost in cultures and disappear after five to six months (unpublished observations). Since G-418-resistant mice (that could be fed with G-418) could not be found, we quantified episomal loss from transfected *Leishmania* cells that contained a small (pTEX vector) or a large (a random cosmid) episome. Two months after infection, amastigotes were recovered from mature lesions and spread onto agarose plates with or without G-418: we found that no more than 50% of the parasites had lost the episome, *i.e.*, could not grow promastigote colonies on G-418 plates compared to control plates (unpublished observations). We considered it was sufficient to interpret results.

Secondly, cosmids contain genomic DNA and genes that could interfere with infectivity. We found predominant cosmids in lesions, and it is possible that they harbored genes that increased the ability of *L. amazonensis* to cause lesions. However, at least for the cosmids we tested, it was not the case. Different clones containing the in vivo predominant T3, T5, T8 or in vitro predominant T15 cosmids displayed different lesion growth rates, showing that these cosmids did not provide an advantage in inducing lesions. This of course cannot be generalised and should be tested systematically.

Our data are compatible with the idea that *L. amazonensis* cell populations consist of a mixture of different cells with different growing abilities in lesions. In vivo growth allowed the most infective ones (the founding cells) to produce lesions. Concomitantly, amastigotes developed different growing potentialities, so that a new diversity of phenotypes was created.

We also observed that in vitro cultivation of promastigotes was not neutral. Cosmid T15 was predominant in 4-month-old in vitro promastigote cultures, while it was not enriched in lesion amastigotes, probably because it was not inside a fast-growing amastigote subclone, or it was not helping for lesion development and was lost (or remained rare) as most of the other cosmids. Why did it become predominant in the promastigote mixture? There are several possibilities: (i) contrary to its amastigote stage, the particular subclone that contained this cosmid grew faster in in vitro culture in its promastigote form than the others, which were progressively eliminated from passage to passage, or (ii) the presence of the cosmid (*i.e.*, of some genes) gave a growth advantage to the parasites in culture medium. Thus, in vitro cultivation of parasites has consequences on the cell population that is kept in culture: only some parasites grow well and tend to predominate; this was to be suspected, but we provide here the beginning of an evidence for this phenomenon. The infectivity changes reported in long-term in vitro cultivation could be interpreted as resulting from the selection of a clonal population which grew well in culture, whether infectious or not. The biological significance of these in vitro changes may be questioned since long-term promastigote cultivation has no real equivalent in natural conditions.

Finally, what has been called infectivity recovery after passage in animals of attenuated parasites (Katakura & Kobayashi 1985) could result from in vivo selection of rare infectious cells. A *Leishmania* cell should be considered as a member of a population with potential phenotypes, rather than an entity with fixed and well-defined characters, and it is the survival of the population, and not of individual cells, that is at stake. As a result, these organisms succeed very well and adapt quickly to rather different natural or artificial environmental conditions.
